# Healthcare utilisation patterns and drivers amongst inflammatory bowel disease patients in the outpatient clinic

**DOI:** 10.1097/MEG.0000000000002880

**Published:** 2024-11-05

**Authors:** Lola J.M. Koppelman, P.W. Jeroen Maljaars, Philip W. Voorneveld, Andrea E. van der Meulen-de Jong

**Affiliations:** 1 Department of Gastroenterology and Hepatology, Leiden University Medical Centre, Leiden, The Netherlands

**Keywords:** care pathways, consultation frequency, healthcare utilisation, inflammatory bowel diseases

## Abstract

**Objective:**

Inflammatory bowel disease (IBD), encompassing Crohn’s disease and ulcerative colitis, impose an escalating burden on healthcare systems globally, with a rising prevalence contributing to increased costs. This study explored healthcare utilisation patterns and its drivers amongst IBD patients in an outpatient clinic.

**Methods:**

A longitudinal cohort study was conducted at a Dutch academic teaching hospital. IBD patients (*n* = 180) were followed for 1 year and were categorised based on disease activity and consultation frequency. Healthcare utilisation was assessed through consultations and laboratory tests. Patient-reported outcomes and biochemical disease activity were measured, and subsequently the reasons for consultations were analysed.

**Results:**

The frequency of outpatient healthcare utilisation exceeded the recommended IBD care guidelines by two-fold. Comorbidities were the leading reason for consultations (40.4%), followed by remission induction, medication changes and pending test results. Moreover, clinical disease activity, reported problems with self-care, daily activities and pain were predictive of an increase in annual consultations.

**Conclusion:**

This study identified factors influencing healthcare utilisation in IBD outpatients. Personalised care pathways using eHealth technologies have the potential to reduce unnecessary consultations and optimise resource allocation.

## Introduction

Chronic diseases pose an ever-growing burden on healthcare systems worldwide. More than forty per cent of the population in the European Union is living with one or more chronic diseases, and this number is expected to grow even further [1,2]. Given this growing prevalence of chronic diseases, high cost of treatment and need for long-term care, healthcare costs will increase [3].

The incidence and prevalence of inflammatory bowel disease (IBD) continue to rise, affecting millions of people worldwide [4]. IBD, including Crohn’s disease and ulcerative colitis, is a chronic inflammatory disease of the gastrointestinal tract characterised by a recurring pattern of disease activity and remission. Currently, there is no curative treatment, and IBD is associated with comorbidities, the need for surgery and prolonged medical treatment [5]. Consequently, this means that IBD-related healthcare utilisation progressively escalates over time [6–9].

As part of the United Nations’ Sustainable Development Goals (Goal 3), all European nations are committed to universal health coverage. To ensure the long-term financial viability of universal health coverage, the European Union Expert Panel on effective ways of investing in health adopted an opinion on defining value-based healthcare (VBHC) [10]. This concept proposes a comprehensive approach based on four fundamental value-pillars aiming at aligning healthcare systems with patient needs and broader societal well-being. These four value-pillars are appropriate care to achieve patients’ personal goals (personal value), achievement of best outcomes with available resources (technical value), equitable resource distribution across all patient groups (allocative value), and contribution of healthcare to social participation and connectedness (societal value). The incentive of this model is to compensate providers such as hospitals and physicians according to patient health outcomes.

Various strategies to reduce healthcare utilisation have been proposed, ranging from IBD-nurse-driven clinics to de-escalation therapy, exit strategies and eHealth technologies [9,11,12]. One of the eHealth technologies available is the care pathway technology delivered by ‘DEARhealth BV’ [13]. DEARhealth offers risk-adjusted care pathway technology for the management of chronic diseases on an Electronic Medical Record (EMR) integrated platform. Biomarkers are used to categorise patients into different risk profiles, each of which corresponds to a different care pathway. Personalised care pathways within the eHealth platform can optimise resource utilisation by promoting standardised evidence-based protocols and guidelines. This supports the technical value pillar of the VBHC model. In addition, DEARhealth can facilitate communication between patients and healthcare providers, allowing patients to easily indicate their health state, and the platform can customise care pathways based on individual patient needs and preferences, ensuring that the treatment aligns with the patient’s goals, maximising personal value.

At the outpatient clinic of two hospitals in Leiden, The Netherlands, the care pathway technology of DEARhealth will be implemented. However, prior to this implementation, it is essential to obtain a better understanding of the current healthcare utilisation of IBD patients at the IBD outpatient clinic. This knowledge can be used to refine and tailor the care pathways to create a more suitable framework. Therefore, this longitudinal cohort study was conducted to investigate healthcare utilisation in the outpatient clinic of patients with IBD in one academic teaching hospital in the Netherlands and to explore the main reasons behind healthcare utilisation.

## Materials and methods

### Study population

Patients aged ≥18 years diagnosed with IBD for at least 3 months were recruited consecutively at the outpatient clinic at the Leiden University Medical Centre between January and May of 2021. The follow-up period was 1 year. At the beginning of the study, patients were classified as low-risk, intermediate-risk or high-risk based on their biochemical disease activity. High-risk patients had ongoing biochemical disease activity [C-reactive protein (CRP ≥5 mg/l) and faecal calprotectin (FCP ≥150 μg/g) individually or in combination], whereas low-risk patients were in biochemical remission for a minimum of 36 months [14]. Intermediate-risk patients were also in remission; however, they had experienced at least one episode of biochemical disease activity within the previous 36 months. Only intermediate-risk and high-risk patients were included at the start of the study. Participants were excluded if they had an ileoanal pouch or an ileostomy. At month 12, biochemical disease activity was reassessed and used to categorise patients into low-risk, intermediate-risk and high-risk groups.

### Outcome variables

Healthcare utilisation in this study was defined by laboratory tests and consultations in an outpatient clinic setting. Outpatient clinic consultations in this study encompassed all appointments conducted by gastroenterologists, nurse practitioners or residents at the IBD outpatient clinic. The consultations included medical e-consultations, medical telephone consultations and live in-person appointments. Administrative calls were excluded from analysis. Outpatient consultations were divided into consultations planned by the healthcare provider and those requested by the patient (by phone, e-mail or e-consultation). The reasons for outpatient clinic consultations were retrospectively scored using the EMR. Each reason was scored once for each patient (independent of the number of consultations). The identified reasons for consultations were routine care, comorbidities (e.g. anaemia, joint problems, perioperative care, pregnancy), remission induction, follow-up due to pending test results from the initial appointment, change in maintenance medication, clarification of additional examination, including clinical trial participation, adverse events, post-admission follow-up, after multidisciplinary consultation (e.g. after doctor-doctor consultation with a surgeon, rheumatologist, gynaecologist, dermatologist, dietician or own colleagues) and counselling for smoking cessation or therapy compliance. If multiple items were discussed during a single consultation, the reason that was filled by the clinician or secretary (in case of patient-initiated consultations) in the EMR in the field ‘reason of consultation’ was scored. When this was unavailable, the researchers determined the primary reason.

At baseline, patients were asked to complete different patient-reported outcomes (PROs) via an online questionnaire. The following information was derived from the EMR: age at inclusion, sex, type of IBD, date of diagnosis, Montreal classification, medication use, indication for colorectal cancer (CRC) screening, history of osteoporosis/osteopenia and laboratory values for CRP, FCP, haemoglobin and ferritin. IBD-related healthcare utilisation at the gastroenterology outpatient clinic during this year was assessed retrospectively using the International Classification of Diseases, 10th Revision codes in the EMR and was categorised into the following subgroups: outpatient consultations (e-consultations, telephone consultations, and outpatient clinic visits), blood draws at the hospital and faecal calprotectin measurements.

#### Patient-reported outcomes

PROs at baseline included a measure of clinical disease activity [mobile Health Index (mHI)], questionnaires about quality of life and health-related quality of life [Short Inflammatory Bowel Disease Questionnaire (SIBDQ) and EQ-6D], and a productivity questionnaire [Work Productivity and Activity Impairment (WPAI) questionnaire] [15–18].

The mHI is a four-item questionnaire developed for remote monitoring of disease activity in IBD patients. This validated questionnaire comes in two forms: mHI-CD and mHI-UC. The mHI-CD score ≥6.38 reflects clinical disease activity in Crohn’s disease (with a negative predictive value of 95%, positive predictive value of 55%, sensitivity of 85% and specificity of 80%), and the mHI-UC score ≥3.20 reflects clinical disease activity in ulcerative colitis (negative predictive value, 95%; positive predictive value, 55%; sensitivity, 85%; and specificity, 80%) [15].

The SIBDQ is a health-related quality of life tool that measures physical, social and emotional status (score 10–70 with higher scores indicating better health-related quality of life) [17].

The EQ-6D is a widely used instrument for measuring health-related quality of life. It considers six dimensions of health: mobility, self-care, usual activity, pain/discomfort, anxiety/depression and cognition. To examine the individual dimensions of the EQ-6D, the categories were transformed into dichotomous variables with the categories ‘no problem’ or ‘any problem’ [18]. The Dutch tariff for the EQ-5D-5L was used to calculate the index score [19]. As the Dutch tariff only exists for the first five dimensions of the EQ-6D, the cognition dimension is disregarded in the index.

The WPAI questionnaire is used to evaluate work productivity loss. The questionnaire comprised four components: percentage of absenteeism (work time missed), percentage of presenteeism (reduced productivity while at work), overall work impairment score that combines absenteeism and presenteeism and activity impairment outside work [16]. All respondents were asked to fill in the activity impairment question, while the other questions were only asked of the employed respondents.

### Scheduled care visits and tests in the inflammatory bowel disease care pathway

**Fig. 1.**
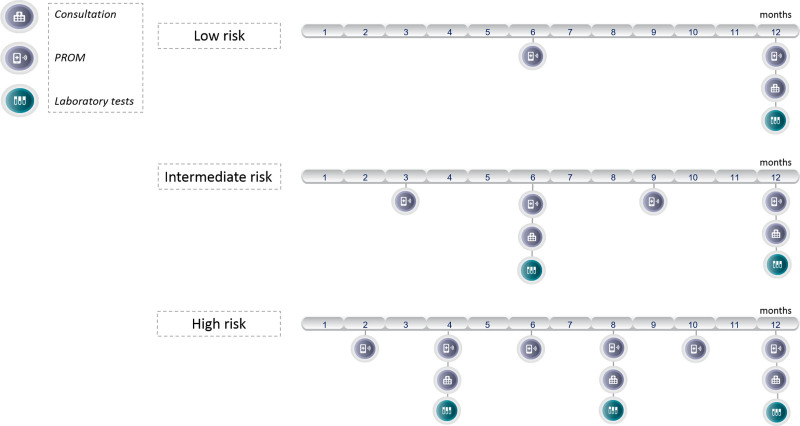
Maintenance care pathways for low-risk, intermediate-risk and high-risk patients. High-risk patients had ongoing biochemical disease activity [C-reactive protein (CRP) >5 mg/l and faecal calprotectin (FCP) >150 μg/g individually or in combination], intermediate-risk patients were in biochemical remission, however they have experienced at least one episode of biochemical disease activity within the previous 36 months and low-risk patients were in biochemical remission for a minimum of 36 months. PROMs include the mHI, EQ-6D, and the WPAI. mHI, mobile Health Index; PROM, patient-reported outcome measure; SIBDQ, Short Inflammatory Bowel Disease Questionnaire; WPAI, Work Productivity and Activity Impairment.

The care pathway technology distinguishes between the three maintenance risk groups (Fig. 1). These care pathways were developed following the latest guidelines and Dutch protocols and have been adapted to the local situation by the company DEARhealth BV together with our own IBD team [20–22]. According to our current maintenance care pathway for IBD patients, low-risk patients require one laboratory test and consultation each year, intermediate-risk patients require two laboratory tests and consultations and high-risk patients require three laboratory tests and consultations each year. Moreover, patients are asked to fill in the mHI. These care pathways serve as a fundamental framework upon which supplementary care can be provided. Three types of supplementary care pathways have been implemented. Patients on immunosuppressive medication undergo biannual outpatient assessments, including blood tests and calprotectin evaluations. Individuals diagnosed with osteopenia or osteoporosis are scheduled for a dual X-ray absorptiometry (DEXA) scan every 3 years. CRC surveillance involves colonoscopies at intervals of every 5 years for low-risk cases, every 3 years for intermediate-risk patients and annually for high-risk individuals. Supplementary care pathways for other reasons, such as comorbidity management and pregnancy of perioperative care, have not yet been included in DEARhealth.

### Statistical analysis

Statistical analyses were performed using SPSS version 29.0 (SPSS Inc., Chicago, Illinois, USA). Continuous variables are presented as mean with SD for normally distributed variables or as median with interquartile range (IQR) for skewed data. Continuous variables were compared using the Mann–Whitney *U* test. Categorical data were compared using the χ^2^ test. Multiple linear regression analysis was used to explore the relationship between healthcare utilisation and clinical disease activity, health-related quality of life (SIBDQ, EQ-6D) and work productivity (WPAI). The dependent variable was log-10 transformed. The model was adjusted for remission induction, hospital admission and additional structured care (e.g. pregnancy, anaemia treatment, joint problems and fistula treatment). Statistical significance was set at *P* < 0.05.

### Ethical consideration

The study protocol was declared not subject to medical research involving human subjects by the Committee on Research involving Human Subjects at the Leiden University Medical Centre. All the patients who participated in this study provided written informed consent.

## Results and discussion

### Study population

The study population consisted of 180 patients (92 patients with Crohn’s disease, 88 patients with ulcerative colitis), and 14 patients were lost to follow-up during the study period of 1 year. The baseline characteristics are shown in Table 1. The median disease duration was 11.0 years (5.8–21.3] with a median age of 41.0 years (30.0–51.0]. Most patients fell into the intermediate-risk category (*n* = 112). Clinical disease activity was notably more severe in the high-risk group than in the intermediate-risk group for both Crohn’s disease and ulcerative colitis. There were marginal differences between the intermediate-risk group and the high-risk group in terms of health-related quality of life and work productivity. However, the health-related quality of life was slightly higher in the intermediate-risk group compared to the high-risk group, and the high-risk group was more impaired during regular activities outside of work (Supplementary Table 1, Supplemental digital content 1, http://links.lww.com/EJGH/B83).

**Table 1.** Baseline characteristics of study population

		Risk groups		
Characteristic	Total (*n* = 180)	Intermediate-risk (*n* = 112)	High-risk (*n* = 68)	*P*-value
Age (years)	41.0 [30.0–51.0]	41.5 [30.0–51.5]	40 [30.0–50.5]	0.777
Sex (female)	92 (51.1)	64 (57.1)	28 (41.2)	0.038
Disease duration (years)	11.0 [5.8–21.3]	11 [6.0–22.0]	12 [5.0–21.0]	0.989
Current smoker	23 (12.8)	17 (16.0)	6 (9.5)	0.232
IBD type (CD)	92 (51.1)	60 (53.6)	32 (47.1)	0.397
Age at diagnosis				0.606
<17 years	36 (20.0)	24 (22.3)	11 (16.2)	
17–40 years	114 (63.3)	69 (61.6)	45 (66.2)	
>40 years	30 (16.7)	18 (16.1)	12 (17.6)	
Disease location (CD)				0.909
Terminal ileum	26 (28.3)	17(28.3)	9 (28.1)	
Colon	17 (18.5)	11 (18.3)	6 (18.8)	
Ileocolonic	48 (52.2)	31 (51.7)	17 (53.1)	
Upper GI tract involvement	1 (1.1)	1 (1.7)	0 (0.0)	
Behaviour (CD)				0.499
Inflammatory	62 (67.4)	41 (68.3)	21 (65.6)	
Structuring	24 (26.1)	14 (23.3)	10 (31.3)	
Penetrating	6 (6.5)	5 (8.3)	1 (3.1)	
Perianal involvement (CD)	20 (21.7)	13 (21.7)	7 (21.9)	0.982
Disease extension (UC)				0.411
Proctitis	13 (14.8)	9 (17.3)	4 (11.1)	
Left-sided colitis	31 (35.2)	20 (38.5)	11 (30.6)	
Pancolitis	44 (50.0)	23 (44.2)	21 (58.3)	
Medication				
No medication	3 (1.7)	3 (2.7)	0 (0.0)	0.174
(local) corticosteroids	39 (21.7)	11 (9.8)	28 (41.2)	<0.001
Amino salicylates^a^	20 (11.1)	18 (22.0)	2 (6.1)	0.042
Thiopurines/methotrexate^a^	15 (8.3)	10 (12.2)	5 (15.2)	0.670
Biologicals/small molecules	117 (65.0)	69 (61.6)	48 (70.6)	0.221
History of osteoporosis/osteopenia	66 (36.7)	42 (37.5)	24 (35.3)	0.766
Surveillance CRC [32]				0.348
No indication	61 (33.9)	41 (36.6)	20 (29.4)	
Low-risk	37 (20.6)	23 (20.5)	14 (20.6)	
Intermediate-risk	58 (32.2)	37 (33.0)	21 (30.9)	
High-risk	24 (13.3)	11 (9.8)	13 (19.1)	
C-reactive protein^b^	2.2 [1.0–6.6]	2.0 [0.9–4.6]	3.1 [1.0–9.0]	0.025
Faecal calprotectin^c^	144.5 [40.0–313.0]	50.0 [19.8–134.3]	269.5 [176.8–1400.3]	<0.001

Data are presented as median (IQR) for skewed data and as *n* (%) for categorical data. Continuous variables were compared using the Mann–Whitney *U* test. Categorical data were compared using the χ^2^ test. High-risk patients had ongoing biochemical disease activity [C-reactive protein (CRP) >5 mg/l and faecal calprotectin (FCP) >150 μg/g individually or in combination], intermediate-risk patients were in biochemical remission; however, they had experienced at least one episode of biochemical disease activity within the previous 36 months.

CD, Crohn’s disease; CRC, colorectal cancer; GI, gastrointestinal; IBD, inflammatory bowel disease; UC, ulcerative colitis.

a With no biologicals/small molecules.

b *n* = 144.

c *n* = 96.

#### Disease activity over time

**Fig. 2.**
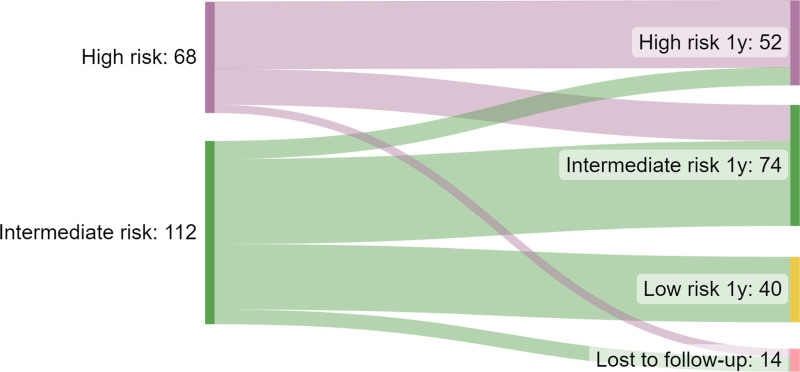
Sankey diagram displaying risk stratification during follow-up. High-risk patients had ongoing biochemical disease activity [C-reactive protein (CRP) ≥5 mg/l andfaecal calprotectin (FCP) ≥150 μg/g individually or in combination], intermediate-risk patients were in biochemical remission, however, they had experienced at least one episode of biochemical disease activity within the previous 36 months and low-risk patients were in biochemical remission for a minimum of 36 months.

After reassessing the disease risk category after 1 year, it was observed that the risk category of the majority of patients (*n* = 93) remained unchanged (Fig. 2). Eleven patients escalated from the intermediate-risk group to the high-risk group, 22 patients de-escalated from high-risk to intermediate-risk and 40 patients de-escalated from intermediate-risk to low-risk.

#### Healthcare utilisation

Healthcare utilisation in the outpatient clinic was analysed in 166 patients. Expected healthcare utilisation was based on the risk stratification assigned at baseline supplemented with the individual surveillance risk for CRC, osteopenia surveillance and medication use. In clinical practice, the high-risk group exhibited a significantly higher healthcare utilisation than the intermediate-risk group (Table 2). To correct for changes in disease activity during follow-up, healthcare utilisation was also compared in patients with a stable disease category throughout the follow-up period. However, aside from patient-initiated contacts, this proved to make no difference (Supplementary Table 2, Supplemental digital content 1, http://links.lww.com/EJGH/B83). When comparing the observed healthcare utilisation to the suggested outpatient care in the maintenance care pathways, IBD patients in this cohort exceeded the recommended level by two-fold. However, these maintenance care pathways do not account for, for example, remission induction therapy, perioperative care or care for pregnancy or joint problems.

**Table 2.** Comparison between healthcare utilisation in the intermediate-risk and high-risk groups

	Intermediate-risk (*n* = 103)		High-risk (*n* = 63)		
	Expected	Observed	Expected	Observed	*P*-value
Outpatient clinic consultations	2.33	4 [3–6]	3.33	6 [5–12]	<0.001
Of which patient-initiated	0	0 [0–1]	0	0 [0–2]	0.037
Frequency of blood draws	2	3 [2–4]	3	5 [3–7]	<0.001
Frequency of FCP test	2	2 [1–3]	3	3 [2– 6]	<0.001

Data are presented as median [IQR]. Mann–Whitney *U* test was used to compare variables. High-risk patients had ongoing biochemical disease activity [C-reactive protein (CRP) >5 mg/l and faecal calprotectin (FCP) >150 μg/g individually or in combination] and intermediate-risk patients were in biochemical remission; however, they had experienced at least one episode of biochemical disease activity within the previous 36 months. Outpatient clinic consultations encompass appointments with gastroenterologists, nurse practitioners or residents and comprise e-consultations, telephone and live in-person appointments. Expected consultations include routine maintenance care complemented by consultations related to colorectal cancer surveillance or osteopenia surveillance.

FCP, faecal calprotectin; IQR, interquartile range.

### Reasons for healthcare utilisation

When exploring the reasons behind consultations in each patient, it was observed that comorbidities, including anaemia, complaints of joint, skin, oral-facial, uveitis, pregnancy and perioperative care, were the main reason for consultations (40.4%), followed by remission induction therapy (23.5%), change in maintenance medication (21.1%) and follow-up due to pending test results from the initial appointment (19.9%) (Table 3). Reasons for consultations in the high-risk group were more often attributed to remission induction therapy, change in maintenance medication, clarification of additional examination, adverse events or consultation after admission to the hospital. For 8.4% of patients, no reason for an additional consultation could be identified.

**Table 3.** Reasons driving healthcare utilisation

		Risk groups		
	Total (*n* = 166)	Intermediate-risk (*n* = 103)	High-risk (*n* = 63)	*P*-value
Routine care	166 (100)	103 (100)	63 (100)	1.000
Comorbidities^a^	67 (40.4)	41 (39.8)	26 (41.3)	0.852
Remission induction	39 (23.5)	10 (9.7)	29 (46.0)	<0.001
Change in maintenance medication	35 (21.1)	8 (7.8)	27 (42.9)	<0.001
Follow-up for pending test results	33 (19.9)	19 (18.4)	14 (22.2)	0.554
Clarification of additional examination	17 (9.4)	6 (5.8)	11 (17.5)	0.016
Adverse event	12 (7.2)	2 (1.9)	10 (15.9)	<0.001
After admission	12 (7.2)	3 (2.9)	9 (14.3)	0.006
Smoking cessation/therapy incompliance	12 (7.2)	6 (5.8)	6 (9.5)	0.372
After multidisciplinary consultation	6 (3.6)	2 (1.9)	4 (6.4)	0.140
Unexplained	14 (8.4)	12 (11.7)	2 (3.2)	0.057

Data are presented as *n* (%). χ^2^ test was used to compare variables. High-risk patients had ongoing biochemical disease activity [C-reactive protein (CRP) >5 mg/l andfaecal calprotectin (FCP) >150 μg/g individually or in combination] and intermediate-risk patients were in biochemical remission; however, they had experienced at least one episode of biochemical disease activity within the previous 36 months. Reasons were scored independent of number of related consultations (1 or 0).

a Including anaemia, complaints of joint, skin, oral-facial, or uveitis, pregnancy, and perioperative care.

To investigate whether PROs for clinical disease activity, health-related quality of life and work productivity and activity impairment (assessed via mHI, SIBDQ, EQ-6D and WPAI, respectively) were predictive of the frequency of annual consultations, a linear regression analysis was conducted. In this model, adjustments were made to account for confounding factors that inherently increase consultation frequency, such as remission induction, hospital admissions and structured care components, such as pregnancy, anaemia treatment, joint issues and fistula treatment. The analysis revealed that clinical disease activity (mHI) was a significant predictor for increased annual consultations (*R*^2^ = 0.389) (Table 4). Furthermore, overall activity impairment, work impairment and problems with daily activities, self-care and mobility contributed to a heightened frequency of consultations. In contrast, higher reported health-related quality of life, measured by both the SIBDQ and EQ-5D-5L index score, were indicative of fewer annual consultations. In examining factors associated with the frequency of patient-initiated consultations, this study revealed that clinical disease activity in ulcerative colitis patients was significantly associated with more patient-initiated consultations. Additionally, this analysis showed that patients who initiated a greater number of consultations tended to have a lower health-related quality of life and a higher incidence of self-care issues when compared to those who initiated fewer or no consultations. However, these findings were not significant and the groups were small (Supplementary Table 3, Supplemental digital content 1, http://links.lww.com/EJGH/B83).

**Table 4.** Multiple linear regression analysis

	Crude β	Adjusted β	*B* (SE)	*R* ^2^	*P*-value
mHI	0.407	0.269	0.159 (0.039)	0.389	<0.001
SIBDQ	−0.318	−0.212	−0.006 (0.002)	0.373	0.002
EQ-6D					
EQ-5D-5L index	−0.277	−0.205	−0.297 (0.094)	0.363	0.002
Mobility	0.128	0.140	0.101 (0.047)	0.342	0.034
Self-care	0.159	0.156	0.219 (0.092)	0.346	0.018
Daily activities	0.222	0.163	0.094 (0.038)	0.348	0.013
Pain	0.257	0.132	0.080 (0.040)	0.339	0.051
Anxiousness	0.139	0.114	0.067 (0.038)	0.335	0.084
Cognition	0.107	0.048	0.029 (0.030)	0.324	0.471
WPAI					
Overall work impairment	0.243	0.169	0.002 (0.001)	0.316	0.044
Activity impairment	0.229	0.226	0.003 (0.001)	0.372	<0.001

Dependant variables are 10-log transformed. Adjusted for remission induction, hospital admission and additional structured care like pregnancy, anaemia treatment, joint problems and fistula treatment. mHI scores for Crohn’s disease (CD) ranges between 0 and 14 (≥6.38 reflects active CD). mHI scores for ulcerative colitis (UC) ranges between 0 and 11 points (≥3.20 reflects active UC).

mHI, mobile Health Index; SIBDQ, Short Inflammatory Bowel Disease Questionnaire; WPAI, Work Productivity and Activity Impairment.

### Discussion

The present study offers a comprehensive exploration of healthcare utilisation patterns amongst IBD patients at the outpatient clinic and highlights the considerable burden of healthcare utilisation within this group, with observed healthcare utilisation surpassing the recommended guidelines [20–22]. Key drivers of healthcare utilisation include comorbidities and contacts related to remission induction, change in maintenance medication and follow-up contacts for pending test results. Moreover, clinical disease activity, and reported problems with self-care, daily activities and pain were predictive of an increase in annual consultations. These insights into the reasons behind healthcare utilisation provide valuable groundwork for optimising patient care strategies and resource allocation.

One area with significant potential for improvement is the follow-up due to pending test results from the initial appointment. As demonstrated, 19.9% of patients required one or more follow-up appointments because of pending test results. Blood sampling post-appointment is common. Patients often travel considerable distances to reach the hospital, leading them to combine biomedical measurements with their outpatient visits. Additionally, some patients undergo blood sampling after their appointment because they forget to do so beforehand. At the studied outpatient clinic, active feedback is not provided following normal test results, as these are available in the patient portal. However, a significant number of phone calls remain necessary due to the implications of test results after the outpatient clinic visits. The integration of personalised care pathways could optimise test scheduling before consultations, enhancing efficiency. These care pathways remind patients of pre-appointment preparations and can prompt healthcare providers to schedule necessary surveillance endoscopies or DEXA scans before outpatient visits. This study showed that 40.4% of the patients could benefit from supplementary care pathways for managing specific comorbidities, including anaemia, joint pain, skin issues and pregnancy. These supplementary care pathways personalise the general maintenance care pathways and include all visits, labs, procedures and virtual monitoring recommended for structured care.

Intermediate-risk patients displayed lower healthcare utilisation compared to high-risk patients. This was underscored by the regression model, which showed a significant association between clinical disease activity and consultation frequency. This finding aligns with existing literature, suggesting that fluctuations in disease activity trigger patient engagement with healthcare services [23–25]. Encouraging the use of PROs, such as the mHI, could reduce patient-initiated consultations by keeping healthcare providers informed about the disease course [26].

It is important to acknowledge certain limitations that may have influenced the findings of this study. The study was conducted during the COVID-19 pandemic, potentially impacting healthcare utilisation patterns with increased consultations related to vaccinations and COVID-19 concerns and reduced in-person visits due to lockdown measures [27]. Part of this effect was corrected by combining telephone consultations, e-consultations and in-person consultations into one category. The retrospective nature of data collection for reasons behind healthcare utilisation introduces limitations. Some consultations lacked clear documentation of reasons, and subjectivity in attributing these reasons may have affected the accuracy of our analysis. The single-centre design in an academic teaching hospital setting, may limit the generalisability of the results to broader IBD populations. Patients in academic settings tend to have a more complex disease courses, which could explain the sizable proportion of consultations for comorbidities [28]. Additionally, the study did not differentiate between appointments with gastroenterologists, nurse practitioners or residents. Despite the well-established benefits of a significant role for nurse practitioners in outpatient settings, assertions about this cannot be made in this study [29].

The findings underscore the potential for optimising care pathways and reducing healthcare utilisation through eHealth technologies, though challenges in implementation, patient engagement and system integration should not be underestimated [30,31]. Balancing personalised care with standardised protocols requires careful navigation. Integrating eHealth technologies, such as the DEARhealth care pathway technology, offer a promising approach to enhance healthcare resource utilisation. These technologies suggest optimal care pathways, potentially reducing follow-up appointments and provide patients the option to inform healthcare providers of changes in disease activity through simple questions, reducing patient-initiated consultations. By personalising care pathways based on individual risk profiles and patient needs, these technologies hold the potential to support the technical value pillar of the VBHC model.

Ensuring healthcare providers are well-trained in using eHealth technologies and integrating them seamlessly with existing healthcare systems, such as automated appointment scheduling in electronic medical records, can improve efficiency. Encouraging the use of digital PROs, such as the mHI, can contribute to the early identification of disease activity, enabling early intervention. Providing patients with comprehensive education and workshops on disease management, symptom tracking and care-seeking, along with eHealth options for routine follow-ups, can further minimise the need for in-person visits. Further research could investigate the long-term impact of these personalised care pathways on healthcare utilisation, patient outcomes and overall healthcare system sustainability.

### Conclusion

In conclusion, our study offers valuable insights into the healthcare utilisation patterns of IBD patients, unveiling the multifaceted factors contributing to consultations. This study contributes to the ongoing efforts in healthcare optimisation.

## Acknowledgements

The authors thank all patients who participated in this study. Additionally, they would like to acknowledge Sara Roozemond for her contributions to the data collection process.

### Conflicts of interest

P.W.J.M. reports consulting fees from AbbVie and Takeda, and payment for presentations from Galapagos and Takeda. A.E.v.d.M.d.J. received research grants from Nestle, Norgine, Cablon, Galapagos and ZonMw, including speaker’s fees from, Tramedico, Janssen Pharmaceuticals, Takeda, Galapagos, Vedanta, Ferring and AbbVie. P.W.V. reports consulting fees from AbbVie, Galapagos, BMS, Janssen, Lilly and Takeda, and payment for presentations from Galapagos and Takeda. These are not related to this manuscript. For the remaining author, there are no conflicts of interest.

## Supplementary Material
