# Supplementary material for: Healthcare utilisation patterns and drivers amongst inflammatory bowel disease patients in the outpatient clinic
Source: Eur J Gastroenterol Hepatol. 2024 Nov 5;37(2):176–83. doi: 10.1097/MEG.0000000000002880 (PMC11658020; doi:10.1097/MEG.0000000000002880)
Supplement: Supplementary file 1 [file ejgh-37-176-s001.pdf]

1 Supplementary data

Supplementary Table 1. PROs

	Risk groups		
	**Intermediate-Risk (n=112)**	**High-Risk (n=68)**	**p-value**
mHI			
Crohn's Disease	2.39 [0.00 – 5.97]	6.20 [4.09 – 8.76]	0.003
Ulcerative Colitis	1.44 [0.00 – 4.24]	4.64 [3.20 – 8.50]	<0.001
sIBDQ	56 [48.5 – 61.0]	52 [42.0 – 60.0]	0.049
EQ-6D			
Index (EQ-5D-5L)	0.852 [0.778 – 0.957]	0.817 [0.704 – 0.887]	0.059
Mobility	19 (54.3)	16 (45.7)	0.259
Self-care	3 (2.9)	5 (7.9)	0.134
Daily activities	47 (44.8)	33 (52.4)	0.338
Pain	63 (57.3)	47 (74.7)	0.054
Anxiousness	39 (37.1)	31 (49.2)	0.125
Cognition	37 (35.2)	26 (41.3)	0.434
WPAI			
overall work impairment	20.00 [10.00 – 40.00]	20.00 [10.00 – 49.29]	0.324
activity impairment	20.00 [10.00 – 40.00]	20.00 [10.00 – 50.00]	0.014

Data are presented as median (IQR) for skewed data. Categorical data are presented as n (). Mann-Whitney U test and. X^2 test were used accordingly to compare variables.

Abbreviations: mHI, mobile Health Index; sIBDQ, Short Inflammatory Bowel Disease Questionnaire; WPAI, Work Productivity and Activity Impairment

High-risk patients had ongoing biochemical disease activity (C-reactive protein (CRP) > 5 mg/L and/or faecal calprotectin (FCP) > 150 µg/g), intermediate-risk patients were in biochemical remission; however, they had experienced at least one episode of biochemical disease activity within the previous 36 months.

mHI scores for Crohn's Disease (CD) ranges between 0-14 (≥ 6.38 reflects active CD). mHI scores for Ulcerative Colitis (UC) ranges between 0-11 points (≥3.20 reflects active UC).

Supplementary Table 2. Healthcare Utilisation in the intermediate-risk and high-risk groups for patients with stable disease activity category during the follow-up period.

	**Risk groups**		
	**Intermediate-Risk (n=42)**	**High-Risk (n=51)**	**P-value**
Outpatient clinic consultations	4 [3 – 6]	7 [5 – 12.5]	<0.001
Of which patient-initiated	0 [0 – 1]	0 [0 – 3]	0.142
Frequency of blood draws	3 [2 – 4]	5 [3 – 8]	<0.001
Frequency of FCP tests	2 [1 – 2]	4 [2– 6]	<0.001

Data are presented as median [IQR]. Mann-Whitney U test was used to compare variables.

High-risk patients had ongoing biochemical disease activity (C-reactive protein (CRP) > 5 mg/L and/or faecal calprotectin (FCP) > 150 µg/g), intermediate-risk patients were in biochemical remission; however, they had experienced at least one episode of biochemical disease activity within the previous 36 months.

Outpatient clinic consultations encompass appointments with gastroenterologists, nurse practitioners, or residents and comprise telephone, e-consultations, and live in-person appointments.

Supplementary Table 3. Association between PROs and patient-initiated contacts

	Number of patient-initiated contacts			
	**0 (n=97)**	**1-3 (n=52)**	**≥4 (n=11)**	**p-value**
mHI				
Crohn's Disease	4.0041 ± 3.4847	4.8201 ± 3.0730	7.3140 ± 3.3993	0.104
Ulcerative Colitis	2.8461 ± 3.0993	4.2635 ± 3.4706	5.5253 ± 2.2048	0.030
sIBDQ	53.8 ± 9.2	50.8 ± 10.7	47.3 ± 11.0	0.077
EQ-6D				
Index (EQ-5D-5L)	0.83 ± 0.15	0.79 ± 0.25	0.68 ± 0.31	0.219
Mobility	17 (17.5)	11 (21.2)	4 (36.4)	0.324
Self-care	3 (3.1)	2 (3.8)	2 (18.2)	0.066
Daily activities	40 (41.2)	26 (50.0)	8 (72.7)	0.112
Pain	61 (62.9)	34 (65.4)	9 (81.8)	0.458
Anxiousness	40 (41.2)	20 (38.5)	6 (54.5)	0.616
Cognition	34 (35.1)	19 (36.5)	5 (45.5)	0.792
WPAI				
overall work impairment	26.9 ± 24.2	34.4 ± 27.4	41.7 ± 19.4	0.093
activity impairment	30.8 ± 22.0	34.8 ± 25.5	45.5 ± 28.4	0.177

Data are presented as mean±S.D. Variables are compared with the Kruskal-Wallis test or X^2 test. Pairwise comparisons using the Dunn-Bonferroni showed no significant results.

Abbreviations: mHI, mobile Health Index; sIBDQ, Short Inflammatory Bowel Disease Questionnaire; WPAI, Work Productivity and Activity Impairment

mHI scores for Crohn's Disease (CD) ranges between 0-14 (≥6.38reflects active CD). mHI scores for Ulcerative Colitis (UC) ranges between 0-11 points (≥3.20 reflects active UC).
